# In-Situ High Resolution Dynamic X-ray Microtomographic Imaging of Olive Oil Removal in Kitchen Sponges by Squeezing and Rinsing

**DOI:** 10.3390/ma11081482

**Published:** 2018-08-20

**Authors:** Abhishek Shastry, Paolo E. Palacio-Mancheno, Karl Braeckman, Sander Vanheule, Ivan Josipovic, Frederic Van Assche, Eric Robles, Veerle Cnudde, Luc Van Hoorebeke, Matthieu N. Boone

**Affiliations:** 1 Radiation Physics Research Group, Department of Physics and Astronomy, Ghent University, Proeftuinstraat 86/N12, B-9000 Gent, Belgium; Sander.Vanheule@UGent.be (S.V.); Frederic.Vanassche@UGent.be (F.V.A.); Luc.VanHoorebeke@UGent.be (L.V.H.); 2 Centre for X-ray Tomography, Ghent University, Proeftuinstraat 86/N12, B-9000 Gent, Belgium; Ivan.Josipovic@UGent.be (I.J.); Veerle.Cnudde@UGent.be (V.C.); 3 Procter and Gamble Corporate Functions, Surface Imaging and Microscopy Department, Mason Business Center, Mason, OH 45040, USA; palaciomancheno.pe@pg.com; 4 The Procter and Gamble Company, Brussels Innovation Center, 1853 Strombeek Bever Temselaan 100, Bever, Belgium; braeckman.k@pg.com; 5 The Procter and Gamble Company, Newcastle Innovation Center, Whitley Road, Longbenton, Newcastle-Upon-Tyne NE12 9TS, UK; 6 Pore-Scale Processes in Geomaterials Research Group (PProGRess), Department of Geology, Ghent University, Krijgslaan 281/S8, B-9000 Gent, Belgium

**Keywords:** X-ray μCT, in-situ experiments, flow cell

## Abstract

Recent advances in high resolution X-ray tomography (μCT) technology have enabled in-situ dynamic μCT imaging (4D-μCT) of time-dependent processes inside 3D structures, non-destructively and non-invasively. This paper illustrates the application of 4D-μCT for visualizing the removal of fatty liquids from kitchen sponges made of polyurethane after rinsing (absorption), squeezing (desorption) and cleaning (adding detergents). For the first time, time-dependent imaging of this type of system was established with sufficiently large contrast gradient between water (with/without detergent) and olive oil (model fat) by the application of suitable fat-sensitive X-ray contrast agents. Thus, contrasted olive oil filled sponges were rinsed and squeezed in a unique laboratory loading device with a fluid flow channel designed to fit inside a rotating gantry-based X-ray μCT system. Results suggest the use of brominated vegetable oil as a preferred contrast agent over magnetite powder for enhancing the attenuation coefficient of olive oil in a multi fluid filled kitchen sponge. The contrast agent (brominated vegetable oil) and olive oil were mixed and subsequently added on to the sponge. There was no disintegration seen in the mixture of contrast agent and olive oil during the cleaning process by detergents. The application of contrast agents also helped in accurately tracking the movement and volume changes of soils in compressed open cell structures. With the in house-built cleaning device, it was quantified that almost 99% of cleaning was possible for contrasted olive oil (brominated vegetable oil with olive oil) dispersed in the sponge. This novel approach allowed for realistic mimicking of the cleaning process and provided closer evaluation of the effectiveness of cleaning by detergents to minimize bacterial growth.

## 1. Introduction

Sponges are ubiquitous implements used in household and industrial cleaning tasks thanks to their flexibility and absorption ability. The cleaning of sponges, i.e., removal of soil on surfaces and cleaning the sponges themselves after absorbing the soil is dependent on the mechanics/hardness of the structure (abrasion level), the interconnectedness and surface area/energy of the open cell structure (capillary forces) [1]. However, the sponge’s complex porous structure may leave soil trapped in the sponge during the sorption phase. Where the soil ends up in the sponge depends on the physical properties of the soil. For example, if it is solid food particles (meat, undissolved carbohydrates, solid fat), it will most likely be deposited on the outer surface of the sponge due to filtration mechanism. However, for liquid soil (vegetable oil, dissolved proteins, dissolved starch) they can be wicked inside the porous structure due to capillary action. These residues whether on the surface or inside the pores may be used by micro-organisms (bacteria, fungi) as a source of food for their growth, giving rise to biofilm formation over time, and hence potential hygiene risks. While detergent formulations with antibacterial active agents may be used to prevent microbial growth in sponges, the use of these antibacterial agents is regulated and limited (EC regulation No 1223/2009 of the European Parliament and of the Council of 30 November 2009 on cosmetic products). Therefore, formulations that are effective in preventing bacterial growth is preferred by ensuring food soils are completely removed inside the sponge. Key to this is the mixture of ingredients in the formulation and the fluid dynamics required for removal. The fluid dynamics inside the complex deformable porous structure are however poorly understood, and therefore their effects on soil removal are not accounted for. To improve the efficiency of cleaning agents, it is important to understand how efficiently these agents are delivered throughout the complex structure of the sponge and to understand the mechanisms that govern the emulsification of the soil inside the sponge. The purpose of this paper is to develop a non-invasive imaging method that can accurately visualize the displacement and relocation of olive oil (or any other animal or plant-based oil) inside an open cell sponge in a multiphase fluid environment and to assess levels of soil removal under compressive forces. Outcomes from this paper can be used as modeling inputs for the evaluation of how sponge absorbs food soil from dishes into the sponge and how these are subsequently removed and released into the wash solution upon squeezing.

In comparison with cleaning sponges (cellulose sponges), kitchen sponges made of polyurethane are less susceptible to tearing because of its high tensile strength [2,3]. Polyurethane sponges are nontoxic and free of biocides unlike cellulose sponges which inherit chemicals to control microbial growth during polymerization process [3]. The sponges have a high water retention capability and release liquid only under pressure [4]. Nevertheless, ester, amide and urethane groups represent sites on the polymer surfaces for hydrolytic attack [5], and are subject to degradation by aqueous acids, alkalis and steam during the cleaning process. This degradation results in structural deformation of the sponge and increase in its overall surface area. The oil sorption capacity increases with the decrease in foam density over time/use and attracts bacterial adhesion. For their commercial availability and known cleaning capacity, polyurethane sponges were studied in this work over other materials.

High resolution X-ray Computed Tomography (μCT) is a non-destructive 3D imaging technique able to visualize both the external and internal structure of porous objects [6]. Throughout the last two decades, it has become an established technique in numerous research areas for visual evaluation of objects. Particularly in porous media, 3D analysis is often performed to extract quantitative parameters like density, porosity, pore size [7,8,9,10]. Also, μCT can be used as input for the generation of 3D geometries which are subsequently used for finite element (FE) [11] or fluid flow simulations [12], and pore scale modeling [13,14,15]. Alternatively, micro magnetic resonance imaging (MRI) can provide dynamic fluid tracking on compressed foam structures; yet, with poor spatial and temporal resolution [16]. In recent years, an increasing interest has extended X-ray imaging to the temporal dimension [17]; exploiting the non-destructive nature of μCT to assess dynamic processes of pores structures such as visualizing multiple phase flow and solute transport in real-time [14,18,19]. Accordingly, μCT was chosen as the ideal technology to track soil removal over MRI. In addition, to achieve temporal analysis of pores structures, in-situ devices have been developed for various purposes in the past including flow cells, and compression stages [20,21]; yet, the application to fluid-filled sponges with soil under dynamic compression has been poorly studied.

An attempt has been made for the imaging of olive oil and its removal from the sponge at a spatial resolution better than 30 microns. However, the possibilities of imaging olive oil in water simultaneously are limited due to the low difference in X-ray attenuating coefficients at the X-ray energies commonly used in μCT [22]. Low contrast driven by small density differences can be countered by the application of X-ray contrast agents which can bind with specificity to the olive oil constituents and enhance its X-ray attenuation against water and PU (Polyurethane). Contrast Enhanced Computed Tomography has been in place for quite some time in medical imaging [23]. However, literature on contrast agents for μCT on inert materials is limited with the research across the globe targeting mainly biological [24], biomedical [25,26,27,28] and geological samples [29,30]. On the other hand, Scanning Electron Microscopy (SEM) staining techniques for polymeric substance can be used for X-ray μCT applications. Staining agents like Osmium (Os) and Ruthenium (Ru) Tetroxide are used to target unsaturated poly-hydrocarbons (e.g., oils and waxes) but unfortunately, both staining agents are toxic and volatile. [31,32,33]. Similarly, phosphotungstic acid (PTA) and phosphomolybdic acid (PMA) can be used to target the conjugated unsaturated fatty oils and proteins. Nevertheless, polyurethane (PU) is not resistant to strong acids and can be hydrolysed [34]. Thus, in this work, application of contrast agents for olive oil inside a fluid filled sponge substrate was accomplished with alternative materials with different physical properties, namely magnetite powder [35] (chemical formula Fe_3_O_4_) and brominated vegetable oil [36]. The contrast is mainly attributed to iron’s and bromine’s higher attenuation coefficient values vs. water, olive oil and polyurethane. Magnetite particles are dispersed in olive oil while brominated vegetable oil is miscible with olive oil. This paper illustrates the application of these contrast agents before and after loading, for the assessment of olive oil removal/cleaning and it is believed to be the first of its kind.

In-situ experiments with X-ray Tomography requires custom build modules capable of mimicking the dynamic process under investigation while respecting the practical constraints of the measurement technique throughout the experiment [37]. In 4D-μCT, the most notable constraints are on the size and composition of the sample holder. Indeed, due to the geometrical magnification in laboratory based μCT, the diameter of the sample should be as small as possible to obtain the desired resolution in the μCT images at maximal flux efficiency (i.e., with a sufficiently small source-to-object distance). In fluid flow experiments, the sample holder completely confines the sample, and should be as transparent as possible to X-rays. Furthermore, a rotational symmetry is desired in tomographic experiments, particularly in the scope of applying corrections for region-of-interest tomography reconstruction [38]. Keeping all these into consideration the flow cell described below was developed to be able to mimic the cleaning process in a realistic way.

## 2. Materials and Methods 

### 2.1. Sample Material

The experiments presented in this paper are conducted on standard kitchen sponges (non-scratch type) made from polyurethane (Spontex, Colombes, France). To improve the reproducibility of the experiments, a large number of sponges was purchased simultaneously, originating from the same production batch. To evaluate the cleaning inside the sponge, they were soiled with commercially available olive oil (Bertolli Extra Virgin, Unilever, Rotterdam, The Netherlands). The cleaning of the sponges was performed using Dreft dishwashing liquid (P&G, Cincinnati, OH, USA) and standard tap water (hard water). For the experiments, sponge samples of 3 cm diameter and 4 cm height were implemented considering the geometrical magnification of X-ray μCT and the required spatial resolution.

### 2.2. Scanner System

In this work, two different high-resolution CT systems were used, both custom-designed by the Ghent University Centre for X-ray Tomography (UGCT, www.ugct.ugent.be). For high quality static imaging of sponges, the HECTOR system was used. This system is based on an open-type XWT240 X-ray tube (X-ray WorX, Garbsen, Germany) and a large PerkinElmer flat-panel detector. More details on the system components can be found in [39]. The best spatial resolution for this system is approximately 4 μm. For the dynamic experiments, the Environmental Micro-CT system or EMCT [40] was used. This gantry-based system is designed specifically to conduct in-situ 4D-μCT experiments, as the source-detector system rotates around the stationary object, thus allowing for several mountings, cables and tubes into the in-situ device, i.e., the flow cell. With a L9181-02 X-ray tube (Hamamatsu Photonics, Hamamatsu, Japan) and a Xineos 1313 flat-panel detector (Teledyne DALSA, Waterloo, ON, Canada), this system is optimized for a compact design and high scanning speed. At highest speed, a full 360° rotation is performed in 12 s, with a best resolution of approximately 15 μm, partly limited by the available X-ray flux. At slower speeds, a best resolution of approximately 5 μm can be achieved. For more information about the scanning system and the relationship between scanning speed and resolution, the reader is referred to [14,40], respectively. Reconstruction of the radiographs obtained during both static and dynamics CT scans was done using Octopus Reconstruction [41] which is an in house developed software package. Octopus Analysis [10] is in house developed software and was used for 3D analysis of the reconstructed images. The 3D rendering of the sponges with the residue was made using VGStudio MAX 3.2 (Volume Graphics GmbH, Heidelberg, Germany).

### 2.3. Flow Cell and Its Automation

**Figure 1:**
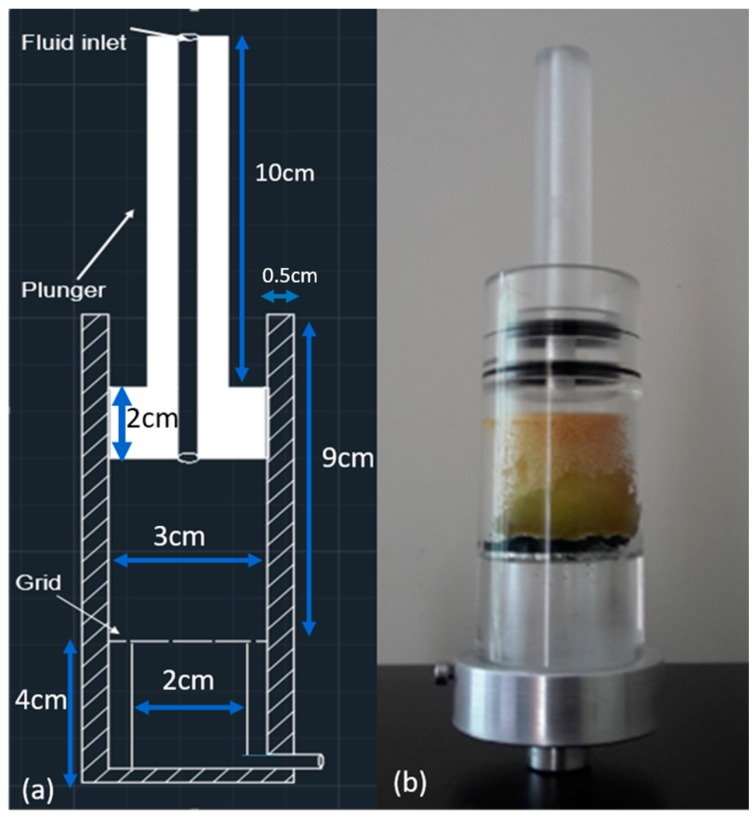
(**a**) 2D front view of CAD model of the sample holder (not to scale), (**b**) photo of the sample holder with sponge.

A sample holder (flow cell) with a provision of a flow channel and the capability to squeeze and flush the sample was designed and constructed in polymethymethacrylate (PMMA). The flow cell consists of a cylindrical body with a grid and a plunger. A grid is placed to hold the sample and drain the fluid out of the sponge upon squeezing. An inlet is provided through the plunger and below the grid a provision is made for the outlet. The tube dimensions are indicated in the Figure 1. The tube can be split into two parts: (1) working area of the tube and (2) region below the grid. The height of the working area is 9 cm with 3 cm inner diameter and the height of the region below the grid plate is 4 cm with 2 cm inner diameter. The height of the plunger is 12 cm, with a protrusion of 3 cm diameter and 2 cm height (Figure 1). The wall thickness of the tube is 0.5 cm and the external diameter of the tube is 4 cm. PMMA was chosen considering the mechanical strength needed for the cyclic action of the flow cell and because of its relatively low X-ray attenuation. The low X-ray attenuation of the flow cell is necessary to make sure that sufficient X-ray flux reaches the detector [42].

**Figure 2:**
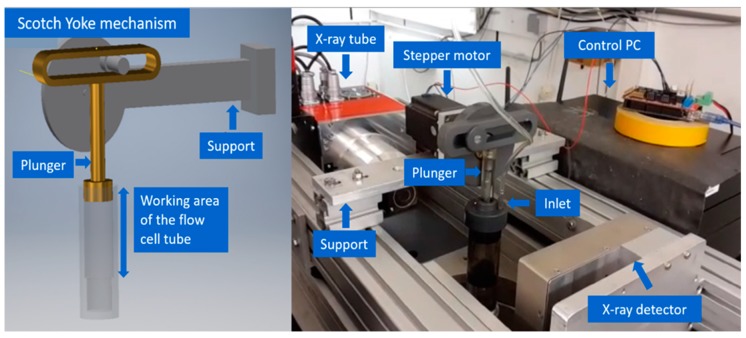
Picture and schematic drawing of the flow cell (Front view) with attached flow channels.

The flow cell can be attached to a scotch yoke mechanism connected to a stepper motor to enable automated squeezing. The supplementary parts are made from PVC and consist of a pinion wheel with a provision to attach the motor shaft, a cap to stabilize the position of the plunger and a support needed to mount the top part of the setup. The total amplitude of the plunging motion is 9 cm. Figure 2 illustrates in detail the add-on modules along with the flow cell. A NEMA23 stepper motor (RepRapWorld B.V, Nootdorp, The Netherlands) with a torque of 30.59 kg.cm is used for providing the thrust required for the reciprocating action of the plunger. The position of the inlet was altered to facilitate easy flow of water through the sponge while squeezing. The flexible hose was attached to both inlet and outlet extensions. The water flow through the flow cell is based on a gravity fed pipe flow system and no electric motor was introduced to pump in water. The outlet was connected to a suitable water basin and the water flow was controlled with a clip attached to the hose.

### 2.4. Experimental Design

Here is a brief overview of all the experiments illustrated in this article.

Preliminary studies:

- Section 2.5 describes two preliminary experiments performed to characterize the samples in more detail. In the first experiment the sponge sample was placed inside the flow cell and fluids (olive oil and water) were added on to the sponge. This system was scanned using X-ray μCT to visualize the microstructure of the sponge and to spot the fluids (water and olive oil) considering their attenuation coefficient values. In the second experiment each of the fluids (olive oil, water and detergent) were scanned separately to determine and note the difference in their attenuation coefficient values.

In-situ test:

- Section 2.7 elaborates on the third experiment where the contrasted olive oil was scanned using X-ray μCT to estimate the attenuation coefficient enhancement of olive oil due to the application of contrast agents. A fourth experiment is also described in which the developed experimental protocol was followed, aiming to demonstrate the cleaning capabilities of the custom-built flow cell and quantification of the contrasted olive oil present in the sponge before and after cleaning (i.e., removal of contrasted olive oil from the sponge) with detergents.

### 2.5. Sample Characterisation

To examine the imaging capability of X-ray μCT for olive oil, water and sponge, preliminary imaging of olive oil in sponges was conducted without the application of any contrast agents on the HECTOR system. For the first experiment the sponge was cut into a cylindrical cross section with a diameter of 3 cm and a height of 4 cm which was placed inside the tube of the flow cell. The flow cell with the dry sponge (sponge without olive oil and water) was scanned using X-ray μCT. Olive oil and water each 5 mL were added to the dry sponge and for 5 min the fluids were allowed to diffuse in the sponge. After this time, the wet sponge (sponge with olive oil and water) was scanned. For both these scans, 1401 projections of 1 s exposure time per projection with a voxel size of 36 × 36 × 36 μm^3^ were acquired over the full 360° rotation. The tube output was set at 70 kV and 30 W and the duration of the scan was around 26 min. The scans were obtained without any use of filters on the X-ray source.

In the second experiment, tubes containing pure olive oil, water and detergent outside the purview of the sponge were scanned using X-ray μCT. Scans with 2001 projections of 1 s exposure time per projection were acquired at full 360° rotation with a voxel size of 37.5 × 37.5 × 37.5 μm^3^ and tube output of 70 kV and 30 W. The duration of the scan was around 36 min. The scans were obtained without any use of filters on the X-ray source.

### 2.6. Contrast Agents

Contrast agents help to improve visualization between the targeted material (i.e., the fatty liquid) and all other constituents of the structure (sponge and detergent solution), including background. In this study, we compare two different contrast agents: magnetite powder and brominated vegetable oil. Commercially available magnetite powder (Inoxia Ltd., Surrey, UK and Natural type) with an average particle size of 40 μm was added to the olive oil at 10% wt/volume. Alternatively, bromine vegetable oil (VWR, Radnor, PA, USA) is another interesting contrast agent which was considered as suitable because it forms miscible solution with olive oil and has higher attenuation coefficient value. The concentration of brominated vegetable oil in olive oil was maintained at 10% wt/volume for the experiments.

### 2.7. Application of Contrast Agents on Sponges

The third experiment aimed at assessing the specificity of each of the contrast agents in olive oil and visualizes the homogeneity of the dispersion (magnetite)/solution (brominated oil). Inside two different containers of 2 cm diameter and 8.5 cm height, 0.5 g of magnetite powder and 0.5 g of brominated vegetable oil were each dispersed respectively in 5 mL of olive oil. Both solutions were observed under X-ray μCT. The tube output for these experiments remained at 80 kV and 12 W. 2401 projections with 0.1 s exposure time per projection were acquired at full 360° rotation at a voxel size of 33.5 × 33.5 × 33.5 μm^3^ in the scan. The duration of scan was around 5 min.

For the fourth experiment the cylindrical cross section of the sponge of 3 cm diameter and a height of 4 cm placed inside the flow cell was first introduced with 5 mL of magnetite powder dispersed olive oil (10% wt/volume) and the following experimental protocol was followed to mimic the soiling and cleaning of the sponge. Later for a new sponge sample, 5 mL of brominated vegetable oil mixed olive oil (10% wt/volume) was introduced and the experimental protocol was repeated.

One compression cycle is the movement of the plunger to its full length inside the working area of the tube and returning to its initial position. The cycle frequency was maintained at ten cycles per minute throughout the whole experiment.

Process 1: The flow cell was connected to an inlet and outlet channel for continuous flow of water through the sponge sample.
Process 2: The sponge was compressed using the plunger for one minute at 10 cycles/min to disperse the contrasted olive oil with continuous flow of water.
Process 3: 5 mL of detergent was added to the dirty sponge and squeezed for one minute at 10 cycles/min inside the flow cell without water supply.
Process 4: At Stage 1 of cleaning process, with live water feed 10 min of squeezing was performed at 10 cycles/min.
Process 5: For the Stage 2 of cleaning process another 5 mL of detergent was added onto the same sponge and again 10 min of squeezing with continuous water feed was performed at 10 cycles/min.
Process 6: In the end the sponge was removed and air dried for a day at room temperature.

At the end of each process the protocol was halted and a μCT scan was acquired using the EMCT system. The duration of the scan was around 5 min each. For these scans, 2401 projections of 0.1 s exposure time per projection with a voxel size of 33.5 × 33.5 × 33.5 μm^3^ were acquired over the full 360° rotation. The tube output was set to 80 kV and 12 W with no hardware filter.

### 2.8. Criteria for Cleaning

The process of cleaning involves squeezing and rinsing of the sponge with the addition of detergent and water to remove the dispersed contrasted olive oil. The volume of the soiled sponge was loaded in Octopus Analysis and by adjusting the threshold the contrasted olive oil present in the sponge was segmented and the volume of contrasted olive oil volume was determined. Considering the same attenuation coefficient for the contrasted olive oil, thresholding was done for the sponge after all cleaning steps. The percentage of cleaning (complementary percentage) (see further in the Section 3.3) was determined by subtracting the ratio of volume of contrasted olive oil determined after cleaning stages to the volume of contrasted olive oil before cleaning from 1 and multiplying the obtained number by 100.

Equation (1)

(1) $$\begin{array}{l} \;\mathrm{Percentage}\;\mathrm{of}\;\mathrm{cleaning}\;\mathrm{for}\;\mathrm{the}\;\mathrm{stage}\;\mathrm{one}\\ \;=\left[1-\;\frac{\mathrm{Volume}\;\mathrm{of}\;\mathrm{contrasted}\;\mathrm{olive}\;\mathrm{oil}\;\mathrm{in}\;\mathrm{stage}\;\mathrm{one}\;}{\mathrm{Volume}\;\mathrm{of}\;\mathrm{contrasted}\;\mathrm{olive}\;\mathrm{oil}\;\mathrm{before}\;\mathrm{cleaning}}\right]\;\\ \;\times 100 \end{array}$$

## 3. Results

### 3.1. Characterization of Materials

**Figure 3:**
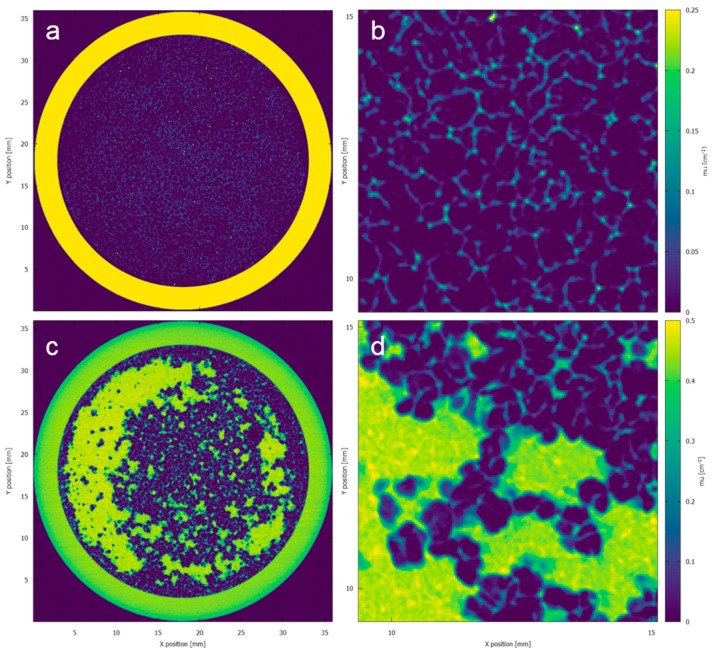
(**a**) Cross sectional image indicating the microstructure of the dry sponge. (**c**) Cross sectional image of sponge with water and olive oil without application of contrast agent. (**b**,**d**) Zoomed images of the result shown in (**a**,**c**), depicting the microstructure of the sponge and water and non-contrasted olive oil in the sponge, respectively. The colormap illustrates the reconstructed attenuation coefficient of different materials.

Figure 3 illustrates the characterization of the sponge microstructure in absence of load, with and without liquids inside. In Figure 3a the pore structure of the dry sponge can be seen and Figure 3c illustrates the distribution of water and olive oil inside the sponge. With the help of 2D cross sectional images the structure and distribution of the fluids at any given layer inside the sponge can be visualized in 2D and 3D. Although the location of olive oil and water inside the sponge could be visually assessed however, in a single system it was impossible to accurately discriminate between olive oil, water and sponge material (Figure 3d).

Table 1 illustrates the experimental attenuation coefficient values of each of the test fluids retrieved separately in multiple scans with same scanner settings. The images were loaded in Octopus Analysis and the average grey value was determined over a volume of interest of approximately 3140 mm^3^. This grey value was later converted to a linear attenuation coefficient μ using the calibration from the reconstruction. Although these values are separated by 2σ (standard deviation), it should be noted that these are obtained in a container of pure material. In a real system such as the sponge, the features to be recognized are small and partial volume effects have a significant contribution [6]. Furthermore, these measurements are obtained from a high-quality scan, and in-situ experiments will yield higher noise levels.

### 3.2. Assessment of Contrast Agent Specificity

In the specificity test, both contrast agents were dispersed evenly in the solution by severe mixing. The magnetite powder solution however proved to be unstable over time due to sedimentation of the powder. The stability of the solution depends on the concentration of magnetite powder dispersed in olive oil. With lower concentrations the onset of sedimentation only happens if the solution remains stationary in its liquid state without being applied onto sponge.

The values of contrasted olive oil in Table 1 indicate that there is a considerable improvement in the attenuation coefficient of olive oil with dispersion of magnetite powder and brominated vegetable oil respectively. 10% wt/volume concentration of contrast agent dispersed in olive oil was chosen to be optimal for all the experiments considering the sedimentation property of magnetite powder in olive oil.

### 3.3. Experiments of Contrasted Olive Oil on Sponges: Cleaning Assessment of the Custom-Built Device and Quantification of Contrasted Olive Oil in the Sponge

Octopus Analysis software was used to calculate the volume of contrasted olive oil present in the sponge at different stages of the cleaning process and it was compared with the actual volume of contrasted olive oil added to the sponge. Although 5 mL of contrasted olive oil was added on to the sponge only 3.6 mL of magnetite powder dispersed olive oil and 1.2 mL of brominated vegetable oil with olive oil could be recorded through image segmentation (Table 2). The factors influencing this difference in value will be explained in the Discussion section.

Using Equation (1) the percentages of cleaning for the sponge were determined. In Table 3 the percentage of cleaning for the given constant volume at two cleaning stages are given. Sponge applied with brominated vegetable oil mixed olive oil showed a better cleaning percentage compared to sponge with magnetite powder dispersed olive oil and the change in the cleaning percentage between the two stages were less for the latter case.

### 3.4. Experiments: Dynamics of Soil Removal from Sponges under Loading

**Figure 4:**
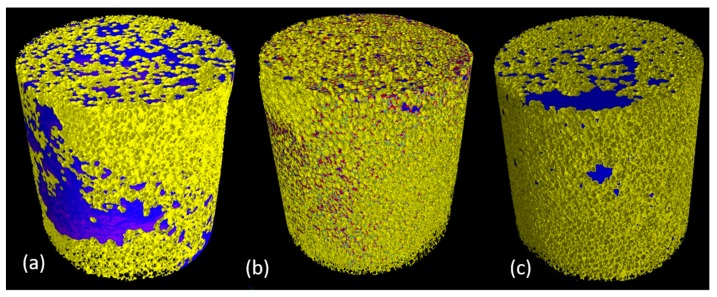
3D rendering of sponge with magnetite powder dispersed olive oil (**a**) before cleaning, (**b**) intermediate Process 3 (Section 2.7) and (**c**) after two stages of cleaning. Pseudo coloration is performed based on the segmentation: blue represents magnetite powder dispersed olive oil, pale blue represents water, red represents detergent and yellow colour represents the sponge. The residue was present even after 2 stages of cleaning for the magnetite powder dispersed olive oil sponge.

**Figure 5:**
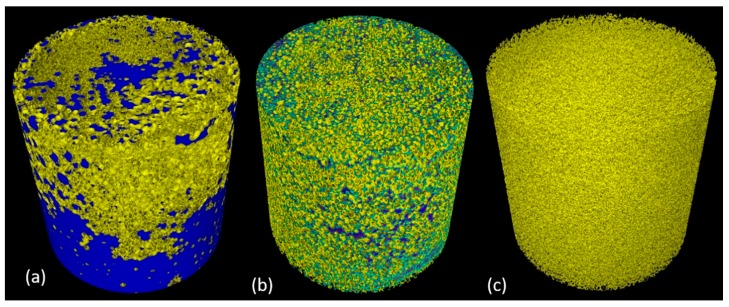
3D rendering of sponge with brominated vegetable oil dispersed olive oil (**a**) before cleaning, (**b**) intermediate Process 3 (Section 2.7) and (**c**) after two stages of cleaning. Pseudo coloration is performed based on the segmentation: blue represents brominated vegetable oil with olive oil, pale blue represents water, red represents detergent and yellow colour represents the sponge. There were no traces of contrasted olive oil in the cleaned sponge.

From the stack of reconstructed slices, a 3D volume of both the uncleaned and the cleaned sponges were rendered using VGStudio MAX 3.2 software. Figure 4 and Figure 5 give the visual representation of the contrasted olive oil with respectively magnetite powder dispersed olive oil and brominated vegetable oil with olive oil in the sponge before and after two stages of cleaning along with the sponge after Process 3 (Section 2.7). The removal of contrasted olive oil in the sponge is made possible by the squeezing action of the plunger, the external compressive force overcomes the capillary forces and thereby the trapped fluid gets displaced by a non-wetting phase. Dynamic action of the plunger not only increases the pore size of the sponge but also helps in mobilizing the water droplets and detergent throughout the porous structure.

## 4. Discussion

Although there is a visible difference between the olive oil and water present in the sponge, it is extremely challenging to quantitatively retrieve the interface between both liquids (Figure 3d). As shown in Table 1, the attenuation coefficient values of these fluids were in close proximity to each other.

The contrast was therefore improved by using two different contrast agents namely magnetite powder and brominated vegetable oil. The dispersion capabilities of the contrast agents in the olive oil and the influence of the contrast agent on the properties of the olive oil are of high importance and limit the practically achievable concentration. Due to the inert chemical behaviour of magnetite powder with olive oil and their difference in bulk densities, sedimentation of magnetite powder occurred at concentrations higher than 50% (wt/volume). For brominated vegetable oil, such sedimentation was not observed as the two liquids were miscible. However, with a thorough premix of the solution, the olive oil acts as a suitable carrier for magnetite powder [43] and the solution remains stable for sufficiently long time before being applied on to the sponge.

With the help of 2D cross sectional images a thin film may be seen on the walls of the sponge pores due to the adhesion of the contrasted olive oil, making it partially a closed cell structure. Also due to the uneven distribution of contrasted olive oil, the sponge tends to lose its stability and collapses towards the heavier side. This was one of the challenges that had to be faced while conducting the experimental protocol. After each step, the sponge was rearranged before the scan to have the full view of the structure.

The traces of magnetite powder dispersed olive oil were present in the sponge even after two stages of cleaning. A possible cause of this phenomenon is the magnetite powder without olive oil which tends to stick to the sponge material due to the heterogeneous solution. This separation of magnetite powder from its solution will not be helpful as the main purpose of cleaning the sponge becomes questionable. The influence of the contrast agent on the olive oil properties, particularly with respect to the interaction with the sponge material, is a very complex research question. Solving this is out of the scope of this manuscript but part of parallel research in the same research consortium. However, the use of brominated vegetable oil as contrast agent for olive oil can be justified as bromine results in a higher attenuation coefficient and the two liquids form a stable solution.

The contrasted olive oil added onto the sponge could impregnate purely by gravity and capillary forces. According to laws of capillarity, the small pores cause higher capillary pressure for the wetting phase (contrasted olive oil) to move towards non-wetting phase (air filled pores) hence making it difficult for imbibition of contrasted olive oil through the sponge. This resulted in the concentration of contrasted olive oil at the periphery (Figure 4a and Figure 5a).

To assess the entire volume of contrasted olive oil present in the sponge, the different phases were segmented in 3D analysis software. The limitation to find the optimal threshold value together with partial volume effects were some of the reasons for the difference between the measured amount of olive oil in the uncleaned sponge and the added amount of olive oil (Table 2, 3.6 mL of magnetite powder dispersed olive oil and 1.2 mL of brominated vegetable oil mixed olive oil compared to 5 mL inserted in the system). One of the other important reasons may be the loss of contrasted olive oil through the outlet after addition, for which solutions will be sought. The amount of solution of brominated vegetable oil in olive oil left behind in the sponge was relatively less and unlike magnetite powder the dissolved brominated vegetable oil did not adhere to the sponge and therefore provides a better approach to contrasting.

As the system was not operated in vacuum, the displacement of the wetting phase by a non-wetting phase resulted in trapping of contrasted olive oil droplets inside the sponge [44]. Dynamic processes like squeezing and rinsing were necessary to mobilize and emulsify this trapped soil droplets for easy removal. Process 2 and Process 3 illustrated in Section 2.7 helped in mobilizing water-soluble particles present in the sponge and in emulsification of fatty soils respectively [45]. These processes facilitated for removal of contrasted olive oil dispersed in the sponge.

The flow cell with its cyclic action and with the help of water channels was successful in removing the contrasted olive oil present inside the sponge. Comparing the percentage of cleaning and the enhancement in attenuation coefficient of olive oil, brominated vegetable oil becomes a favorable choice as contrast agent. The 3D rendered images in Figure 4 and Figure 5 depict the displacement and relocation of fluid inside the porous structure in relation to time and hence can be used as an input for developing a model for fatty oil absorption and removal with a detergent solution. This method can be employed to optimize a detergent formulation that can quickly wet the kitchen sponge and the oil trapped in between the pores, emulsify the fat into oil droplets and completely remove them via squeezing action.

X-ray 4D-μCT (time dependent imaging) is a suitable choice for visualizing in situ experiments as it needs no sample preparation and gives both qualitative and quantitative data. Application of suitable contrast agents have further enabled the technique to image low attenuating samples in a multiphase fluid system non-destructively. 

## 5. Summary

A 4D-μCT approach to assess removal of fatty oil from sponge has been developed. A sample holder with a flow channel and the capability to squeeze and flush the sample was designed to follow the dynamic processes inside the sponge during oil removal. Two contrast agents were assessed to improve visualization of the fatty oil absorption, emulsification and removal. Magnetite particles provided contrast but the stability of the magnetite dispersion in olive oil and the magnetite adsorption onto the sponge inner surfaces were identified as the major problems with this approach. On the other hand, mixing brominated vegetable oil with olive oil was identified as the better approach as it forms a solution with olive oil. This fatty oil solution does not phase separate and does not adsorb strongly to the sponge inner surfaces thus behaving like olive oil. Washing the sponge with a dishwashing solution almost completely removed the oil indicating deep down cleaning and can help minimize bacterial growth inside the sponge.

## Figures and Tables

**Table 1:** Solvents with their experimental linear X-ray attenuation coefficient (μ) and the achieved standard deviation σ (μ) indicating the close proximity in attenuation coefficient values of test fluids and attenuation coefficient enhancement of olive oil by addition of contrast agents.

Solvents	μ (cm^−1^)	σ (μ) (cm^−1^)
Olive oil	0.25	0.0158
Water	0.33	0.0261
Detergent (Dish washing liquid)	0.35	0.0297
Magnetite powder dispersed olive oil	0.45	0.0421
Brominated vegetable oil with olive oil	0.62	0.0775

**Table 2:** Volume of contrasted olive oil at different stages of cleaning indicating the removal of contrasted olive oil from the sponge. Stage 1 of cleaning process: soiled sponge thoroughly rinsed with 5 mL of detergent is squeezed for 10 min at 10 cycles/min with continuous water feed. Stage 2 of cleaning process: 5 mL of detergent is added to the same sponge and squeezed for 10 min at 10 cycles/min with continuous water feed.

Contrasted Olive Oil	Actual Volume of Contrasted Olive Oil	Uncleaned Sponge	Stage 1	Stage 2
Volume of magnetite dispersed olive oil (mL)	5	3.6	0.8	0.79
Volume of brominated vegetable oil with olive oil (mL)	5	1.2	0.08	0.01

**Table 3:** Percentage of cleaning for two sponges, sponge 1 (magnetite powder dispersed olive oil) and sponge 2 (brominated vegetable oil mixed olive oil).

Sponge Samples	Stage 1 of Cleaning	Stage 2 of Cleaning
Sponge 1	77.2%	77.5%
Sponge 2	92.5%	98.8%
